# Tears as a Source of Biomarkers in the Diagnosis of Graves’ Orbitopathy

**DOI:** 10.3390/biom12111620

**Published:** 2022-11-02

**Authors:** Diana Bajkowska, Małgorzata Szelachowska, Angelika Buczyńska, Adam Jacek Krętowski, Katarzyna Siewko

**Affiliations:** 1 Department of Endocrinology, Diabetology and Internal Medicine, Medical University of Bialystok, 15-276 Bialystok, Poland; 2 Clinical Research Centre, Medical University of Bialystok, 15-276 Bialystok, Poland

**Keywords:** Graves’ orbitopathy, tear fluid, biomarkers, proteomics, cytokines, ocular pathologies, precision medicine, prognostic

## Abstract

Thyroid eye disease (TED) is a poorly understood autoimmune manifestation of thyroid diseases most commonly associated with Graves’ disease. Due to a lack of specific biomarkers and uncertain signs and symptoms, Graves’ orbitopathy (GO) is challenging to diagnose early and treat effectively. Nowadays, there is great interest in searching for precise molecular biomarkers for early detection, disease monitoring, and clinical management. Researchers are keen to identify novel methods to predict and diagnose diseases and to monitor patient therapeutic response. Tears, due to their direct contact with the eye and the fact that lacrimal glands can also be affected by the disease, could give new insights into the mechanisms taking place in thyroid-associated orbitopathy (TAO) and reveal potential promising biomarkers. Tear fluid offers the possibility of the non-invasive acquisition of a sample with a high protein content, thereby attracting continuously growing interest in the discovery of novel biomarkers. This article provides an up-to-date overview of the various putative tear-fluid biomarkers that have been identified. In this review, we present the potential use of tears as a diagnostic fluid and tool to investigate the mechanism of ocular diseases and discuss the future research directions in this area.

## 1. Introduction

Graves’ ophthalmopathy/orbitopathy (GO), also known as thyroid eye disease (TED), is the most common extra-thyroid manifestation of Graves’ disease (GD). Unambiguous identification of the factors underlying Graves’ orbitopathy has not yet been accomplished [1,2]. Genetic, epigenetic, and environmental factors have been suggested to contribute to the development of thyroid-associated orbitopathy (TAO). There is a close temporal relationship between the onset of GO and the onset of hyperthyroidism. In approximately 85% of cases GO and hyperthyroidism occur within +/−18 months of each other [3]. In addition, GO can also arise in patients with euthyroidism or hypothyroidism due to autoimmune thyroiditis. Thus, the concept that GO is not directly provoked by the abnormal thyroid hormone levels associated with GD is now widely accepted. Rather, experts believe that TAO occurs as the consequence of underlying autoimmune processes [2,3,4,5]. 

The autoimmune process involving the tissues surrounding the eyes is poorly understood, though it is known that antigens of both orbital and thyroid tissues exhibit cross-reactivity. Orbital fibroblasts express receptors, such as thyroid-stimulating hormone receptor (TSHR) and insulin-like growth factor-1 receptor (IGF-R1). In response to the activation of these receptors by autoantigens, fibroblasts may modulate immune responses via the production of cytokines and extracellular matrix/hyaluronan and elicit an inflammatory reaction in the orbit. The production of glycosaminoglycans (GAGs) by orbital fibroblasts and the hyperplasia of adipose tissue contribute to the development of ocular symptoms such as enlarged extraocular muscles and increased fatty and connective tissue, resulting in protruding eyes, double vision, and in severe cases, visual field loss or even loss of vision [1,4,6,7,8,9]. The symptoms of GO are primarily due to inflammation, and the severity of GO is categorized as mild, moderate, and severe (including sight-threatening) [10,11].

The detection of TED is based on clinical signs and symptoms and is largely subjective, with no established biomarkers able to differentiate TED from GD alone [12].

Nowadays, researchers are keen to identify novel methods to predict and diagnose GO and to monitor patient therapeutic response. The advancement of technology is facilitating the quantification of minute samples that were previously difficult to assay [13]. Thus, researchers are starting to focus on how the autoimmune and inflammatory reactions that take place in the orbit of GO patients affect the proteomic and metabolomic composition of tear film. Tear collection is less invasive compared with orbital tissue or blood serum. Human tear fluid is a complex mixture comprising water (98%), more than 1500 proteins that could be diagnostically relevant, lipids, electrolytes, mucins, metabolites, hormones, and desquamated epithelial cells, as well as foreign substances from the ambient air. In this context, tears, due to their direct contact with the eye and the fact that lacrimal glands can also be affected by the GO, could provide new insights into the mechanisms taking place in TAO disease and have potential as promising biomarkers. To date, numerous biomarker candidates have been identified to play a role in GO; however, little is known about the function of most tear components [13,14]. In this context, many studies have quantified the amount of proteins and cytokines in tears and have found several differences in active GO, inactive GO, and healthy controls [7]; in this review, we focus on the most recent finding. This literature aimed to answer the following question: What is the role of tear fluid in the diagnosis of TAO?

## 2. Current Recommendation for TAO Diagnostic

There are no unambiguous criteria for the diagnosis of GO. Diagnosis is based on symptoms, ocular signs, and results of laboratory and imaging tests. The complete ophthalmic examination includes the clinical activity score (CAS), slit-lamp examination, refraction, visual acuity, intraocular pressure, ocular motility, fundus photography, Hertel exophthalmometry, palpebral fissure width, and computed tomography [1,15]. Magnetic resonance imaging of the orbit is useful in distinguishing extraocular muscle enlargement from fat expansion. Especially in cases of asymmetric proptosis, exclusion orbital tumor, and arterio–venous malformation is important [16]. In each case, TSH, fT3, and fT4 determinations should be assessed. It is also very important to test antibodies against TSH-R because a high concentration indicates unfavorable prognostic for the course of orbitopathy [17]. Unfortunately, there is currently no specific biomarker available that can differentiate TED from GD or mild TED from moderate-to-severe TED. Better tools to more precisely diagnose this severe complication are warranted [1,12]. 

### Diagnosis of Tears vs. Orbital Tissues

To expand diagnosis, the majority of studies conducted to date on the pathogenesis of GO have focused mostly on orbital tissues. This method is invasive, and samples are only available from patients through surgery, which are a limitation. Contrastingly, tear collection is a less invasive technique. The advantage of the tear sampling method is that it is more accessible, easy to collect, easy to obtain permission from patients, triggers less pain, and can allow repeated measurements over time [1].

## 3. Markers in the Tears of Patients with Graves’ Orbitopathy

### 3.1. Cytokines and Chemokines in the Tears of Patients with Graves’ Orbitopathy

Cytokines, including interleukins (ILs), tumor necrosis factors (TNFs), interferons (IFNs), lymphokines, and chemokines, are small proteins important in normal physiology and in host responses to infection, trauma, reproduction, inflammation, sepsis, and tumors [18]. As mentioned above, GO is associated with immune and inflammatory processes. Cytokines likely play a crucial role in the development of the disease process by participating in the induction and effector phases of immune interactions between T and B cells, macrophages, orbital fibroblasts, and orbital adipocytes [13,19,20]. For example, IL-1 and TNF-α stimulate intercellular adhesion molecule 1 (ICAM-1) expression and glycosaminoglycan production in orbital fibroblasts [21]. Thus, there are a great number of studies focused on identifying the profiles of cytokines and chemokines within tears that are characteristic of the different clinical subgroups of patients with GO.

Following a study performed by Kishazi et al. [22], the levels of some cytokines, such as IL-10, IL-12p70, IL-13, IL-6, and TNF-α, were found to be significantly higher in the tears of TAO patients than those of healthy subjects. Interestingly, IL-10, IL-12p70, and IL-8 levels increased in tears regardless of the form of TAO, whereas IL-13, IL-6, and TNF-α levels were significantly elevated in inflammatory TAO patients, meaning those with a clinical score activity (CAS) ≥ 3, compared with controls. It was also shown that none of the cytokines exhibited sufficient differences to allow patients with the mild form of TAO to be distinguished from control subjects. However, patients with the mild form of TAO could be discriminated from control subjects on the basis of sIL6-R. In this study, Kishazi et al. [22] compared their results with those of previous studies. For example, in two separate studies, Huang et al. [23,24] observed the upregulation of IL-6 and IL-8 in TAO patients in addition to some discordant results. For example, TNF-α concentrations were significantly higher in both active and inactive TAO compared with the controls, and the upregulation of IL-1β and IL-17A in reflex tears was also observed [23,24].

Several studies have focused on cytokines that allow active and inactive GO to be distinguished. For example, Cai et al. [25] suggested that IL-7 may play an important role in GO pathogenesis. They compared patients with active and inactive GO and healthy controls on the basis of tears and orbital tissue. As found using ELISA, patients with inactive GO had the highest IL-7 concentrations in tears, followed by healthy controls and patients with active GO. Immunohistochemistry analysis showed that IL-7 expression in the orbital tissues of the inactive GO samples was higher than that of the volunteers. These results have been confirmed in other studies [19,23]. Huang et al., in one study, showed that IL-7 was highest in inactive TAO when compared with active TAO and the healthy group, and in another, that IL-2 levels were significantly higher in inactive TAO compared with the controls [23,24]. Yang et al. [19] isolated cytokines specific to active GO in the tears of affected patients. Of all the examined cytokines, only IL-7 yielded a difference regarding concentrations in patients with active GO. Low concentrations of IL-7 in tears exhibited specificity for active GO in patients nearly two years after the clinical onset of activity. Although the use of IL-7 in tears as a biomarker for disease activity may be limited due to its late manifestation, knowledge of the impact of IL-7 in GO may have disease-modifying effects. Moreover, the production of IL-15 and IL-17 was higher in inactive GO patients than in active GO patients, suggesting that IL-15 and IL-17 are linked to TAO pathogenesis and development [26].

Due to the lack of established biomarkers that allow for differentiating TED from GD alone, Ujhelyi et al. [27] investigated the levels of seven cytokines and plasminogen activator inhibitor-1 (PAI-1) in tear samples of GD patients with GO, patients with GD but without GO, and healthy controls. They found significantly higher release of IL-1β, IL-6, IL-13, IL-17A, IL-18, TNF-α, and RANTES in GO patients compared with controls, but no significant differences were found in cytokine release between the GO and GD groups. In the GO group, a significant positive correlation was found between CAS and the release of IL-6 and PAI-1 into tears. IL-6 release into tears was also found to be correlated with both CAS and the degree of eyeball protrusion. PAI-1 release was significantly higher in GO than in GD patients and was increased in both the GD and GO groups compared with controls. Ujhelyi et al. [27] wanted to characterize the cytokine profile of tears in patients with Graves’ disease with and without orbitopathy. Unfortunately, none of the cytokines was able to distinguish these two groups. Prior to their study, no comprehensive non-targeted proteomic study had been conducted based on a highly relevant comparison between GD patients with and without GO. Therefore, although such a study has now been successfully completed due to this report on PAI-1, more studies in this direction are needed.

In another extended study performed by Song et al. [28], authors evaluated the tear proteomics profile among patients with Graves orbitopathy compared to healthy volunteers. The received results showed that 10 protein concentrations (CD40, CD40 ligand, GITR, IL-12p70, IL-1 beta, IL-2, IL-21, IL-6, MIP-3 alpha and TRANCE) were upregulated and 3 protein concentrations (GM-CSF, IL-1 sRI, and IL-13) were downregulated in GO patients. The limitation of this study was a small plurality of research and control group (14 patients summary).

Concerning recent studies, some authors were interested in tear inflammatory cytokines and ocular surface changes in patients with active TAO treated with high-dose intravenous glucocorticoids [29]. They analyzed seven inflammatory cytokines (IL-1β, IL-6, IL-8, IL-10, IL- 17A, TNF-α, and VEGF) and noticed significantly elevated levels in active TED patients compared with controls, except in the case of IL-17A [29]. This was in contrast to previous reports, wherein increased levels of IL-17A were noted [23,24,27]. Compared with the baseline, concentrations of IL-1β, IL-6, IL-8, TNF-α, and VEGF were significantly decreased following 12 weeks of effective treatment. Significant positive correlations were found between IL-6, IL-8, and CAS. IL-6 increased in active TED compared with inactive TED after glucocorticoid treatment. Moreover, a negative correlation was found between IL-6 levels and TED duration before methylprednisolone treatment [29]. Furthermore, those results indicate, for the first time, that tear inflammatory cytokines decrease following treatment with systemic glucocorticoids. However, that study also had some limitations. A placebo group was lacking, and some patients were excluded from the study because they did not respond adequately in four weeks, and therefore, the levels of cytokines were not checked in these patients [29]. Cytokines have been considered a powerful tool for identifying novel biomarkers in GO. Due to the fact that immune activity in orbitopathy is neither synonymous nor coincident with the clinical severity of eye disease, researchers are still seeking correlations between tear inflammatory mediators and clinical parameters. In the discussed works, a significant positive correlation was found between CAS and the release of IL-6, IL-8, IL-13, and TNF-α into tears. Further clinical investigations of these biomarkers, namely, regarding their specificity and sensitivity, may point toward the selection of suitable biomarkers for aiding in the detection and prognosis of TED. In future research, studying additional groups of Graves’ disease without orbitopathy and after systemic treatment could also be clinically pertinent [22,27,28,29,30,31] (Table 1).

### 3.2. Proteomic Markers in Tears of Patients with Graves’ Orbitopathy

The proteome describes the protein component expressed in cells and tissues. By using proteomic techniques, isoforms and protein post-translational variants can also be evaluated [32]. Many researchers have found evidence for the altered composition of tears in TAO. Hence, in recent years, the proteomic analysis of tear fluids from patients with TAO has been undertaken to better determine disease activity and to stratify patients accordingly, because the high concentration of proteins in tear fluid makes it an important source for studying potential protein biomarkers for GO [13,33,34]. Many studies have found several differences in the amount of proteins in active GO, inactive GO, and healthy controls, so we focused on the most recent findings [7].

An interesting study by Matheis et al. [35] reportsed on the proteomic profiling of tear fluid in patients with autoimmune thyroid eye disease (Graves’ disease or Hashimoto’s thyroiditis) of various degrees of clinical severity and clinical activity and a control group, using SELDI-TOFMS and SDS-PAGE. Peptides with molecular weights of 3808, 3734, and 3837 Dalton (Da) were identified as proline-rich protein 4 (PRP4) and downregulated in patients with TAO versus controls. The abundance of the 3837 Da peptide was negatively correlated with the clinical activity score and age. The higher the CAS values and the degree of inflammation, the lower the PRP4 levels, probably indicating progressive inflammation-induced dysfunction of the lacrimal gland in TAO. Moreover, a 12,003 Da peptide was identified as ß2-microglobulin and decreased in tear fluid as the clinical severity of TAO increases. In contrast, a 5815 Da peptide identified as lysozyme C increased with clinical severity. Between treated and untreated patients with TAO, an 11,770 Da peptide was upregulated and identified as cystatin S, which probably reflect a beneficial effect of steroids. The same group of scientists [35,36], in 2015, identified a protein panel allowing significant differention between GO patients versus dry eye and/or controls. They found an upregulation of inflammatory proteins versus a downregulation of protective proteins in GO [35,36]. Lysozyme C and lactoferrin were also upregulated in tear samples obtained from patients with active period TAO compared with age- and gender-matched healthy subjects [37].

In the study of Jiang et al. [38], involving a proteomic analysis comparing GO patients and healthy controls, albumin and C3 levels were significantly lower in the tears of active GO patients than in volunteers, and the author speculated that these proteins may be involved in the pathogenesis of GO. During the last year, the same group of authors realized that most of these tear proteomics studies have focused on the active stage or mixed types in TAO, so they identified significantly changed proteins when comparing inactive-stage GO patients with healthy controls. Proteomic profiling identified 62 upregulated and 45 downregulated proteins in the inactive stage of TAO compared with healthy individuals. The signaling pathways of the immune system, apoptosis, cell cycle, metabolism of carbohydrates, protein synthesis, degradation, and transport may play key roles in patients with inactive TAO [39].

Aass et al. [34] compared the tear fluid of GD patients who had moderate/severe GO with that of patients who had GD without GO (controls) using untargeted quantitative proteomics and identified differences in 1212 proteins. Among them, 16 proteins, including lysozyme C (LYZ), lacritin (LACRT), and zinc-alpha-2-glycoprotein (AZGP1), were significantly upregulated in GD patients who had moderate/severe GO compared with GD patients lacking ocular involvement [34]. One year later, their aim was to assess individual levels of LYZ, LACRT, and AZGP1 in tears from GD patients with moderate-to-severe GO and those without GO. The tear levels of LYZ, LACRT, and AZGP1 were significantly elevated in GD patients who had moderate-to-severe GO compared with GD patients without GO after adjusting for age, smoking, and gender. LYZ levels were also measured in tears from GD patients with mild GO and without GO. Significantly higher levels of LYZ were measured in GD patients with mild GO than in those without GO [10]. Thus, a novel three-protein biomarker panel was established as a result. It remains to be elucidated whether proteins can be used as markers for patients at risk of developing GO, as well as as useful indicators for disease activity.

Concerning recent studies, other groups have also identified these three proteins (lysozyme C, lacritin, and zinc-alpha-2 glycoprotein) but with nonsignificant TAO/control ratios [14]. The reason is probably the difference between the types of control groups chosen. Kishazi et al. [14] examined tears in TAO patients and healthy controls. They found upregulated cystatin C and serpin A3. This is fully in line with the cytokine level increases that they observed in their next study [22]. IL-6 is one of the major regulators of serpin A3, found to stimulate its synthesis and secretion. Concerning cystatin C, its expression has been shown to be upregulated by TNF-α but decreased by IL-10. The fact that cystatin C was found to be upregulated despite the upregulation of IL-10 may imply that the power of TNF-α to promote upregulation exceeds that of IL-10 to downregulate cystatin C expression. This duality may also explain why cystatin C was found with a lower upregulated ratio in TAO patients compared with serpin A3. The overall conclusion of the two parts of the study was that the inflammatory process appears to be the main pathway deregulated in TAO disease and could be, at both cytokine and protein levels, a very promising target for the improved management of patients. Significantly higher alpha-1-antichymotrypsin levels were observed in patients with CAS ≥ 3 than in those with CAS < 3. Retinal dehydrogenase 1 was found to be downregulated in the tears of TAO patients in comparison with the tears of control subjects. Aass et al. also studied this protein but found no significant differences in the protein levels between GD patients with or without orbitopathy [34].

Proteomic results are constantly changing, showing how sets of proteins interact with environmental factors [40]. Furthermore, the concentrations of many proteins depend on their locations in biological compartments and the phase of the cell cycle, which can also be interrupted by many diseases [41,42]. In the results described above, we see differences depending on the selected research group and control group. Hence, in proteomics studies, the unification of the group of respondents is required. Most of the presented studies had the same group of patients, but they differed in two cases. For example, in the study by Aass et al., the control group did not comprise healthy patients but consisted of patients with Graves’ disease without GO. Proteomic research focusing on differences between GD and TAO is still limited. In the most recent studies, scientists have focused on mild orbitopathy in contrast to the earlier focus on moderate to severe GO. In future studies, introducing more groups, such as of patients with Graves’ disease without orbitopathy and with mild orbitopathy, could be clinically pertinent.

In a study from last year, a group of researchers sought to identify proteins differentially expressed between thyroid-associated orbitopathy (TAO) and GD in serum. In the results, differentially expressed proteins (DEPs) were systematically identified in patients with TAO, GD, and healthy controls. Most of the DEPs were involved in metabolism, inflammation, macromolecule biosynthesis, and single-organism cellular processes. A total of 46 proteins showed a significant change in GD patients vs. the healthy control group, 110 in GD vs. TAO, and 136 in TAO patients vs. the healthy control group. This study had the limitation of investigating only a small group of patients, namely, nine (three from each group), although it does provide new insights and potential targets for studying GD with TAO [43]. It should be emphasized that proteomics has already contributed to the significant progress made in determining which biological pathways are adversely affected in Graves’ orbitopathy (Table 2).

### 3.3. Oxidative Stress Markers in Tears of Patients with Graves’ Orbitopathy

Oxidative stress is represented by excess reactive oxygen species (ROS), which disturb the intracellular redox state, leading to peroxide and free radical accumulation and subsequent damage to proteins, lipids, and DNA in cells. The involvement of oxidative stress in the pathogenesis of GD and GO has been examined throughout the years [44]. Various markers of oxidative damage have been identified. Among these, 8-hydroxy-2′-deoxyguanosine (8-OHdG) and malondialdehyde (MDA) are the most commonly investigated by-products of DNA oxidation and lipid peroxidation [45]. Increased levels of extracellular ROS have been noted in the fibroadipose tissues, blood, orbital fibroblasts, and urine of GO patients [32,46,47].

In a study of participants with inactive-stage GO, active-stage GO, and healthy controls without GO, oxidative stress markers were investigated in the tears of patients with Graves’ orbitopathy. The study showed, first, that, compared with the control, increased levels of 8-OHdG and MDA were observed in the tear films of patients with GO, especially those with active-stage GO. Second, the levels of the 8-OHdG and MDA were positively correlated with CAS and reflected disease severity in the active-stage GO group [1]. In the study, the scientists confirmed the reduction of MDA in the serum of patients with GO who had been treated with glucocorticoids [48].

In future studies, it would be worthwhile to quantify the level of ROS in tears after treatment with glucocorticosteroids.

### 3.4. Metabolomic Profiles as Graves’ Orbitopathy Markers

Among the metabolomics methods, nuclear magnetic resonance (NMR) spectroscopy and mass spectrometry (MS) are mainly used [49]. Metabolomics methods are frequently suggested as having immense potential for early diagnosis, simultaneously leading to the better understanding of the pathogenesis of many diseases. Thus, knowledge of a patient’s tear metabolome can extend the diagnostic potential of TAO. Following a study performed by Billiet et al. [50] using mass spectrometry, wherein the accurate measurement of 44 metabolites to compare the metabolic composition of tears in patients with active versus inactive TAO was performed. Two short-chain acylcarnitines, propionylcarnitine and butyrylcarnitine, and spermine were reported with increased concentrations in the tears of patients with active TAO. In contrast, ornithine, glycine, serine, citrulline, and histidine showed decreased concentrations in this group. Accordingly, the pathophysiological model of the tear metabolic signature were obtained in active versus inactive TAO, wherein the increased spermine synthesis was connected to active TAO. This was the first metabolomic study that examined the tears of patients with TAO, which resulted in novel insights in this field for the future studies. Metabolomic methods for biomarker discovery are characterized by high sensitivity and specificity, which also leads to the discovery of novel TAO medical targets.

## 4. Graves’ Orbitopathy and Dry Eye Syndrome

Dry eye disease (DED) is a multifactorial disease of the ocular surface characterized by a loss of homeostasis in the tear film, accompanied by ocular symptoms, in which tear-film instability and hyperosmolarity, ocular surface inflammation and damage, and neurosensory abnormalities play crucial roles in pathogenesis [51]. Dry eyes are a common symptom in patients with TAO. Eckstein et al. [52] found that significant ocular surface damage was correlated with reduced tear secretion, and these researchers were the first to demonstrate that lacrimal acinar cells physiologically express TSHR. This suggests that, in thyroid disease, autoantibodies may bind to lacrimal TSHR and, perhaps via aberrant signal transduction, contribute to LG impairment and, hence, dry eye syndrome [52].

In comparative studies, tear-film osmolarity was significantly higher in patients with TAO than in healthy subjects. Suggested reasons for this are the increased width of the palpebral fissure, incomplete blinking, and increased proptosis that causes ocular surface drying and tear hyperosmolarity (evaporative dry eye), which results in subsequent ocular surface damage [24,53].

Following the many similarities observed between dry eye and orbitopathy syndrome symptoms for specific markers, differentiation is still needed. In the tear samples obtained from patients with TAO, DED, a combination of TAO + DED, and controls, proteomics profile analyses were performed by Matheis et al. [36]. In this study, 18 protein levels significantly differed between the TAO and the dry eye patient groups. Proline-rich protein 1 (PROL1), uridine diphosphate (UDP)–glucose-dehydrogenase (UGDH), calgranulin A, transcription-activator BRG1, annexin A1, cystatin, heat shock protein 27, and galectin were downregulated in TAO compared to the DED group. Lysozyme C was upregulated in TAO versus TAO + DED. Thus, the proteome results showed a different protein panel in patients with TAO and those with dry eye syndrome, which is potentially useful in tear TAO vs. DED screening [36]

Nowak et al. [54] found that dry eye symptoms were present in 85% of patients with thyroid ophthalmopathy compared with 30% in the control group, so the use of artificial tear drops and modifications of environmental conditions should be recommended in such patients [54].

Dry eye syndrome in TAO could result in problems with collecting tears due to reduced volumes, which should be considered during the analysis of results.

## 5. Limitation of the Novel Diagnostic Tears Methods Introduction

Considering tears as a material useful in TAO routine clinical management and diagnosis, several limitations were also observed. Nowadays, omics methods, such as metabolomics and proteomics, are mainly placed in the scientific field; thus, the costs of the diagnostic kit and equipment are still expensive. However, omics methods could be incorporated in everyday routine diagnostic use as costs for sample analyses continue to decrease, which has been noticed with technological progress. Nevertheless, the data analysis required professional programs, databases, and experienced personnel, which have to complete a lot of courses and training. However, the omics-received diagnostic properties are highly recommended due to more specific and personalized diagnosis. Since omics methods could be incorporated into routine clinical management, a great advance in TAO diagnostics could be achieved.

## 6. Conclusions

Based on our literature search, it can be concluded that tear analysis demonstrates vast potential for TAO. These studies support the idea that tears reflect biological modifications occurring in a disease context and can therefore be a promising fluid for biomarker discovery. The benefits of tear fluid collection lie in the availability, noninvasive collection, and lower variability in the components compared with blood serum or plasma [26,34,51]. A comprehensive examination of tears makes it possible to understand the pathophysiology and mechanism of GO because of the proximity of the inflammatory process and the frequent involvement of the lacrimal gland in TAO. The identification of suitable biomarkers can allow their use as a diagnostic tool for the earlier detection of TAO and the differentiation of TED from GD alone and mild TED from moderate to severe TED, which is necessary due to the largely subjective detection of TED based on clinical signs and symptoms. 

Studies have linked the presence of various cytokines in tears with disease activity. Thus, the cytokines and the signaling pathways they activate represent attractive therapeutic targets. The interruption of these pathways might alter the natural course of Graves’ disease and its orbital manifestations [55]. The application of proteomic technologies in the evaluation of biological compartments creates novel possibilities for elucidating the pathomechanism and discovering novel drug targets and early disease markers. 

In summary, by determining the compositional changes in tear profiles, crucial pathways in disease progression may be identified, allowing more predictive and personalized therapy for individuals [13]. However, there is still a need to provide useful data in order to validate their usefulness from being used in clinic diagnostics. We believe that the potential of tear diagnostics would be particularly useful in routine clinical management.

## Figures and Tables

**Table 1:** Summary of studies examining cytokines in tears of GO patients.

Aim of the Study	Method of Collection	Number of Patients	Tear Sample Amount Collected	Group of Patients	Main Findings	Tear Analysis Technique	References
To measure the levels of 10 cytokines: interferon-γ (IFN-γ), interleukins: IL-10, IL-12-p70, IL-13, IL-1β, IL-2, IL-4, IL-6, IL-8, tumor necrosis factor-α (TNF-α), and soluble interleukin-6 receptor (sIL-6R)	Schirmer paper strips	20 TAO patients and 18 healthy controls	Mean wetting of 19.5 ± 4.3 mm was obtained for healthy control subjects compared with 19.1 ± 5.9 mm for TAO patients in 3 min.	TAO patients vs. healthy controls and TAO inflammatory patients vs. controls	IL-10, IL-12p70, IL-13, IL-6, and TNF-α were significantly higher in TAO patients than in healthy subjects. IL-13, IL-6, and TNF-α levels were significantly elevated in inflammatory TAO patients compared with controls. sIL-6R levels could discriminate between patients with a mild form of TAO and control subjects.	10-plex panel (Meso Scale Discovery Company) and Invitrogen Human sIL-6R ELISA kit	Kishazi et al., 2018 [22]
To explore tear inflammatory cytokines in (TAO) patients	2 μL microcapillaries	24 TAO patients and 16 age-matched normal control subjects	6 μL of tears was obtained from each patient.	Two TAO groups (active and inactive) and the control	Concentrations of IL-1β, IL-6, IL-17A, and TNF- α were significantly higher in TAO patients than in the control subjects. IL-7 was highest in inactive TAO among the three groups.	MILLIPLEX Human Cytokine/Chemokine Panel [MPXHCYTO-60K]	Huang et al., 2014 [23]
To investigate the levels of seven inflammatory cytokines and one chemokine in tears of patients with TAO and asymptomatic control subjects	2 μL microcapillaries	21 patients with TAO and 10 asymptomatic controls	About 10 μL of tears was obtained from each patient.	Two TAO groups (active and inactive) and the control	IL-1β, IL-6, and IL-8 were significantly higher in active TAO than in inactive TAO and the controls. TNF-α concentration was significantly higher in active and inactive TAO vs. controls. IL-17 was significantly higher in active TAO than in the controls. The level of IL-2 was significantly higher in inactive TAO compared controls.	Luminex system	Huang et al., 2012 [24]
To explore whether IL-7 participates in the pathogenesis of Graves’ ophthalmopathy (GO)	10 μL microcapillaries	20 GO patients and 20 healthy volunteers	About 10 μL of tears was obtained from each eye.	Active GO vs. inactive GO vs. healthy control	Patients with inactive GO had the highest IL-7 concentrations in the tears followed by healthy controls and patients with active GO	ELISA	Cai K et al., 2013 [25]
To isolate cytokines specific for active GO	Schirmer paper strips	10 patients with active GO and 10 patients from each of 3 control group (30 controls)	x	Active and inactive GO with the 3 control groups (normal female patients, patients with inactive GO, and patients with bilateral viral conjunctivitis)	Low concentrations of IL-7 exhibit specificity for active GO. IL-1B and IL-6 in the active and inactive groups were higher compared with the normal group, but there were no differences between the active and inactive groups.	BCA protein assay and Bio-Plex Human Cytokine 17-plex panel	Yang et al. 2018, [19]
To investigate the levels of IL-15 and IL-17 in GO patients (active and inactive stages) and healthy volunteers	x	24 active GO, 24 inactive GO patients, and 24 healthy volunteers	20 μL tears from both eyes	Active GO patients and inactive (after GKS treatment) compared with healthy controls	In comparison with the volunteers, the significant upregulation of IL-15 and IL-17 was identified in active and inactive GO patients. Compared with inactive patients with GO, the levels of IL-15 and IL-17 in the tears were obviously higher in the active patients with GO.	ELISA	Chen Q et al., 2019 [26]
To characterize the cytokine profile of tears in patients with Graves’ disease (GD) with and without GO	Capillary tubes	27 GO patients, 9 GD patients, and 12 controls	x	GO patients with GD patients and with controls	IL-1β, IL-6, IL-13, IL-17A, IL-18, TNF-α, and RANTES were significantly higher in GO patients compared to controls. A significant positive correlation was found between CAS and the release of IL-6 and PAI-1 into tears. PAI-1 release was significantly higher in GO than in GD and was increased in both the GD and GO groups compared to controls.	FlowCytomix Simplex and Basic Kit	B. Ujhelyi et al., 2012 [27]
To explore the cytokine profile in tears of patients with Graves’ ophthalmopathy	x	7 patients with active GO and 7 healthy volunteers	x	Active GO vs healthy volunteers	10 protein concentrations (CD40, CD40 ligand, GITR, IL-12p70, IL-1 beta, IL-2, IL-21, IL-6, MIP-3 alpha, and TRANCE) were upregulated, and 3 protein concentrations (GM-CSF, IL-1 sRI, and IL-13) were downregulated in GO patients.	High-throughput protein microarray technology	Song et al., 2020 [28]
To evaluate high-dose intravenous glucocorticoid treatment on tear inflammatory cytokines and ocular surface parameters in patients with active TED	2 μL microcapillaries	15 moderate-to-severe and active patients and 15 healthy subjects	The volume of a single tear sample was 10 μL.	Moderate-to-severe active TED pre and post-treatment and control subjects	Levels of all baseline cytokines except IL-17A were significantly elevated in active TED patients compared with controls. Concentrations of IL-1β, IL-6, IL-8, TNF-α, and VEGF were significantly decreased at 12 weeks of treatment compared with the baseline. There were significant positive correlations between IL-6, IL-8, and CAS, and a negative correlation was found between the IL-6 level and TED duration before methylprednisolone treatment. The reductions of IL-6, IL-8, and VEGF were positively correlated with the reduction in CAS at 12 weeks.	Human Magnetic Luminex Performance Assay	Xu N et.al., 2020 [29]

vs., versus; x—no information mentioned.

**Table 2:** Summary of Studies Examining Proteins in Tears of GO Patients.

Aim of the Study	Method of Collection	Number of Patients	Tear Sample Amount Collected	Group of Patients	Main Findings	Tear Analysis Technique	References
To characterize the protein profile of tears in patients with autoimmune thyroid eye disease of various degrees of clinical severity and CAS and control group	Schirmer paper strips	45 patients with TAO and 15 healthy controls	x	TAO patients with autoimmune–thyroid disease (GD or Hashimoto’s thyroiditis) vs. healthy controls and treated vs. untreated patients with TAO	PRP4 were downregulated in TAO patients vs. healthy controls. ß2-microglobulin was downregulated in TAO patients compared with controls and decreased in tear fluid with increased clinical severity of TAO. Lysozyme C was upregulated in TAO patients and increased with clinical severity. Cystatin C was upregulated in treated patients with TAO vs. untreated.	SELDI-TOF-MS technology	Matheis et al., 2012 [35]
To explore tear inflammatory and protective proteins in GO patients	Schirmer paper strips	60 TAO patients with various degrees of CAS, 30 DED patients and 30 healthy controls	x	GO patients vs. dry eye and/or healthy controls	Inflammatory proteins were upregulated, and protective proteins were downregulated in GO patients. Significantly different protein panel in TAO versus dry eye and/or controls.	MALDI-TOF/TOF mass spectrometer	Matheis et al., 2015 [36]
To explore the differential expressions of lysozyme C and lactoferrin in tears of TAO patients versus healthy subjects by proteomics	x	x	x	Active TAO compared with age- and gender-matched healthy subjects	Lysozyme C and lactoferrin were upregulated in TAO patients’ tears than in the controls.	x	Jiang et al., 2015 [37]
To compare tear fluid contents of GO patients with that of healthy subjects	10 μL capillary glass tubes	25 active GO patients and healthy controls	A total of 5 μL of tears was obtained from each participant.	GO patients vs. healthy control	Serum albumin and C3 levels in tears of active GO patients were significantly lower than in the tears of healthy volunteers.	SDS-PAGE	Jiang et al., 2013 [38]
To identify significantly changed proteins of TAO with the late, inactive stage	10 μL capillary glass tubes	Six TAO patients with CAS score < 3 and six control healthy subject	A total of 5 μL of tears was obtained from each participant.	Patients with a CAS score < 3 (inactive GO) compared with the control group	Proteomic profiling identified 107 significantly changed proteins between the inactive stage of TAO patients and healthy cases. 62 were upregulated, and 45 were downregulated in inactive TAO cases compared to healthy individuals.	LC-MS/MS	Jiang et al. 2021, [39]
To quantitatively compare tear fluid proteins from GD patients with moderate/severe GO and patients with GD without GO (controls)	Schirmer paper strips	21 patients with moderate to severe GO and 21 controls	x	GD patients with moderate/severe GO and patients with GD without GO (controls)	A total of 16 proteins, including lysozyme C (LYZ), lacritin (LACRT), and zinc-alpha-2-glycoprotein (AZGP1), were significantly upregulated in GD patients who had moderate/severe GO compared to GD patients without GO.	LC-MS/MS	Aass et al., 2016 [34]
To evaluate tear levels of LYZ, LACRT, and AZGP1 in GD patients with or without GO	Schirmer paper strips	21 patients with moderate to severe GO, 21 GD with no clinical signs of GO (controls); another study group: 18 patients with mild GO and 9 patients without GO	x	GD patients with moderate/severe GO and patients with GD without GO (controls). GD patients with mild GO and without.	The tear levels of LYZ, LACRT, and AZGP1 were significantly elevated in GD patients who had moderate to severe GO compared to GD patients without GO. Significantly higher levels of LYZ were measured in GD patients with mild GO than in those without GO.	ELISA	Aass et al., 2017 [10]
To investigate the tears of the TAO patients in order to identify potential biomarkers	Schirmer paper strips	28 TAO patients and 25 healthy controls	x	TAO patients and healthy controls	The cystatin C and serpin A3 were upregulated. Significantly higher alpha-1-antichymotrypsin levels were observed in patients with CAS ≥ 3 than in those with CAS < 3. Retinal dehydrogenase 1 was found to be downregulated in tears of TAO patients in comparison with the tears of control subjects.	SDS_PAGE, LC-MS/MS, ELISA, Western Blood	Kishazi et.al., 2018 [14]

vs., versus; x—no information mentioned.

## Data Availability

Not applicable.

## Abbreviations

The abbreviations including in the text are reported alphabetically

**Abbreviation**	**Meaning**
AZGP1	zinc-alpha-2-glycoprotein
CAS	clinical activity score
Da	Dalton
DED	dry eye disease
DEPs	differentially expressed proteins
GAGs	glycosaminoglycans
GD	Graves’ disease
GO	Graves’ orbitopathy
ICAM-1	intercellular adhesion molecule 1
IFNs	interferons
IGF-R1	insulin-like growth factor-1 receptor
ILs	interleukins
LACRT	lacritin
LYZ	lysozyme C
MDA	malondialdehyde
MS	mass spectrometry
NMR	nuclear magnetic resonance
PAI-1	plasminogen activator inhibitor-1
PROL1	proline-rich protein 1
PRP4	proline-rich protein 4
RANTES	regulated on activation, normal T-cell expressed and secreted
ROS	reactive oxygen species
TAO	thyroid associated orbitopathy
TED	thyroid eye disease
TNFs	tumor necrosis factors
TSHR	thyroid-stimulating hormone receptor
UGDH	uridine diphosphate (UDP)–glucose-dehydrogenase
VEGF	vascular endothelial growth factor)
8-OHdG	8-hydroxy-2′-deoxyguanosine
